# Supplementary material for: Conserved signalling components coordinate epidermal patterning and cuticle deposition in barley
Source: Nat Commun. 2022 Oct 13;13:6050. doi: 10.1038/s41467-022-33300-1 (PMC9561702; doi:10.1038/s41467-022-33300-1)
Supplement: Supplementary file 1 — Supplementary Information [file 41467_2022_33300_MOESM1_ESM.docx]

**Supplementary Note 1**

**Construction of the double mutant *cer-s.31* and *cer-g.10***

Crossing the two mutants gave a wt F_1_ that was allowed to self. Eight resulting *cer* F_2_ plants were backcrossed to the two *cer* mutant parents and also allowed to self. A double mutant was not found however four F_2_s were found that exhibited a 1:1 ratio of *cer*:wt phenotype with the cross to one parent and only *cer* plants with the other indicating the F_2_ was homozygous for the mutant allele at one of the two genes and heterozygous for the other. Selfs of these F_2_s were sown out and phenotypic selection allowed the identification of the double mutant and selfed seeds collected from this F_3_ plant. The homozygosity for mutant alleles at both loci was confirmed by subsequent crosses to Bonus which gave 100% wt progeny (n≈400) and to either parent (*cer-s* or *cer-g*) which gave 100% *cer* progeny in both cases (n≈400).

**Linkage analysis**

To test for linkage the double mutant was crossed to Bonus (wt) which gave a phenotypically wt F_1_. If the loci were not linked, selfing the F_1_ would yield a 9 wt:7 *cer* ratio in the F_2_; however a ratio of 1338: 659 (wt : *cer*) was observed i.e. 67%: 33% which indicates linkage (χ2 = 93.79, p < 0.001). If the loci *cer-s* and *cer-g* are 12 map units apart then the expected proportion of *cer* individuals is 32.92% (see Punnett square below) giving expectation of 1339.6 wt: 657.4 *cer*.

|  | ++ (0.44) | mm (0.44) | +m (0.06) | m+ (0.06) |
| --- | --- | --- | --- | --- |
| ++ (0.44) |  |  |  |  |
| mm (0.44) |  | 0.1936 *cer* | 0.0264 *cer* | 0.0264 *cer* |
| +m (0.06) |  | 0.0264 *cer* | 0.0264 *cer* |  |
| m+ (0.06) |  | 0.0264 *cer* |  | 0.0036 *cer* |

Punnett square giving expected frequencies of phenotypically *cer* F_2_ individuals from a cross in coupling between two linked *cer* loci 12 map units (13.5 cM) apart. (+ = wt and m= mutant allele at the two loci).

The linkage found agrees with previous studies that have mapped both loci (*Cer-s* = *Gsh5* and *Cer-g*) to the long arm of chromosome 2H (2HL) ^1,2^. To our knowledge no previous study has estimated the linkage directly between these loci, though a number of studies have demonstrated linkage of these *Cer* loci to common morphological markers ^1,2^ and with standard translocation tester lines ^3^. There are more reports of linkage studies involving *Cer-s* (*Gsh5*) with linkage found to *Vrs1* ^4^ on 2H with recombination values found ranging from 22.07% -32.50% ^5-7^. Linkage studies involving *Cer-g* indicate that it is also linked to *Vrs1* with an estimated recombination value of 15.9 ^6,8^. Given the relative recombination values to additional marker loci, the position of *Cer-s* is estimated to be proximal to *Vrs1* whereas *Cer-g* is distal ^1,2^ implying that *Cer-s* and *Cer-g* are potentially only loosely linked on 2HL (24.2 % *Cer-s* -*Vrs1* ^6,7^ and 15.9 % *Vrs1 – Cer-g* ^6^) unlike the tighter linkage found here using the mutants in coupling.

The identification of *Cer-s* (HORVU.MOREX.r2.2HG0144720) and *Cer-g* (HORVU.MOREX.r2.2HG0155920) and of *Vrs-1* (HORVU.MOREX.r2.2HG0152930) ^4^ indicates that the linear order of the three genes is as expected. In addition, the physical position of the genes also allows the *Cer* loci to be placed in silico on the SNP map derived from genotyping a Golden Promise x Morex RIL population (that segregates for *vrs1*) with the 50K SNP array ^9^. The positions of *Cer-s*, *Vrs1* and *Cer-g* on chromosome 2H would thus be at 63.28, 75.39 and 82.42 cM respectively meaning *Cer-s* and *Vrs1* would be 12.11 cM apart, *Vrs1* and *Cer-g* 7.03 cM apart and *Cer-s* and *Cer-g* 19.14 cM apart consistent with the tighter linkage found in this study. Given the physical locations of the loci on the Morex v2 genome (*Cer-s* 533,631,062; *Cer-g* 599,988,582) the loci are 66.4 Mbp apart, corresponding to 3.5 cM per Mbp in the RIL population, whereas in the present study using the mutants in coupling it corresponds to 4.9 Mbp per cM.


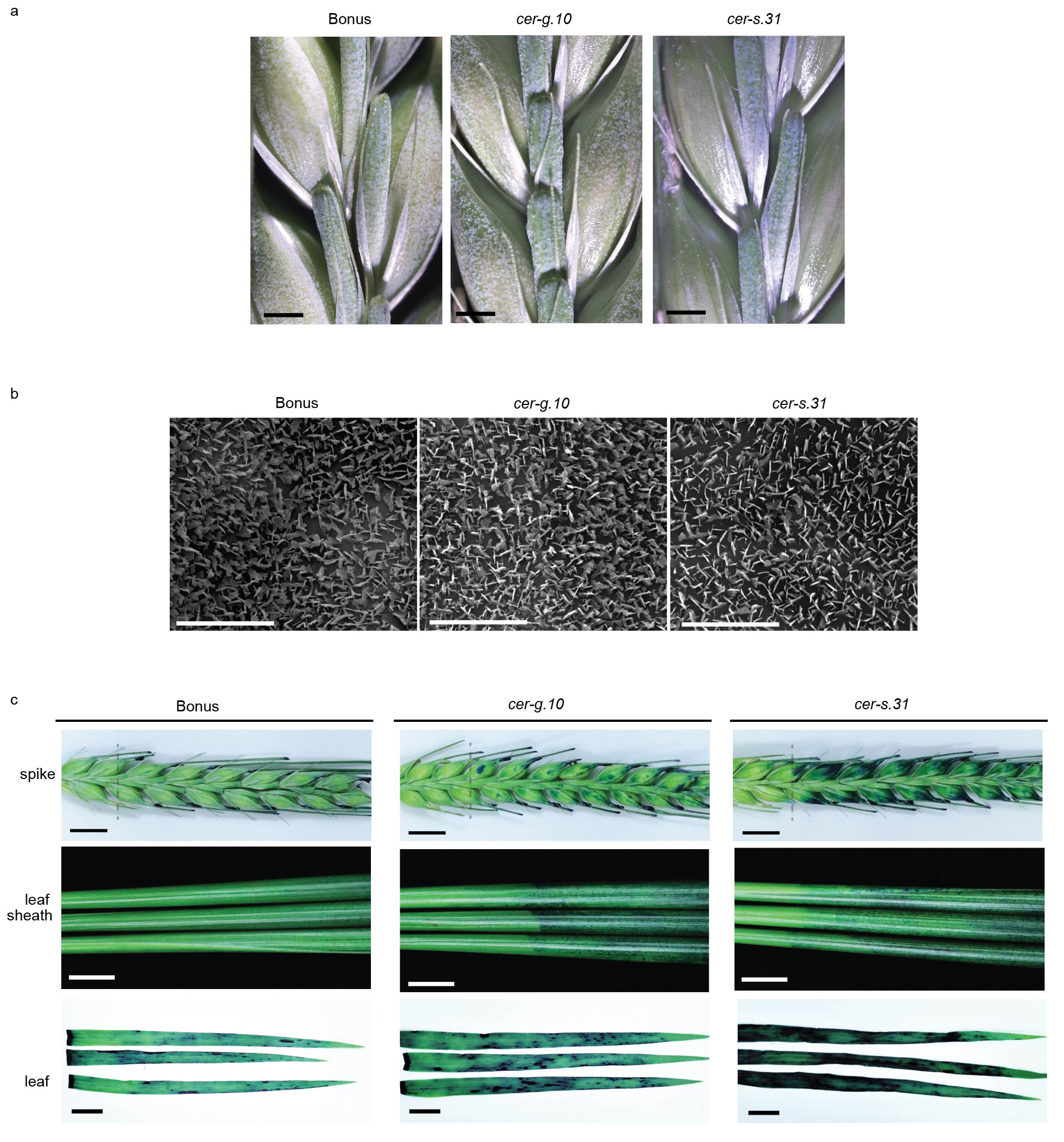


**Supplementary Fig.1 Cuticle defects of *cer-g.10* and *cer-s.31***. **a**, sections of whole spikes from Bonus, *cer-g.10* and *cer-s.31* (scale bars = 2mm). **b**, representative images of epicuticular wax crystals on abaxial side of the first leaf of 14 day-old of Bonus, *cer-g.10* and *cer-s.31* (10,000× magnification, scale bars = 10 µm). N = 4 plants per genotype. **c**, representative photos of toluidine blue staining of Bonus, *cer-g.10* and *cer-s.31* spikes, leaf sheaths and second leaves. Tissues to the right of the dashed vertical lines were submerged in toluidine blue for 5 h (spikes), 28 h (sheaths) and 24 h (leaves). Scale bars = 1cm.


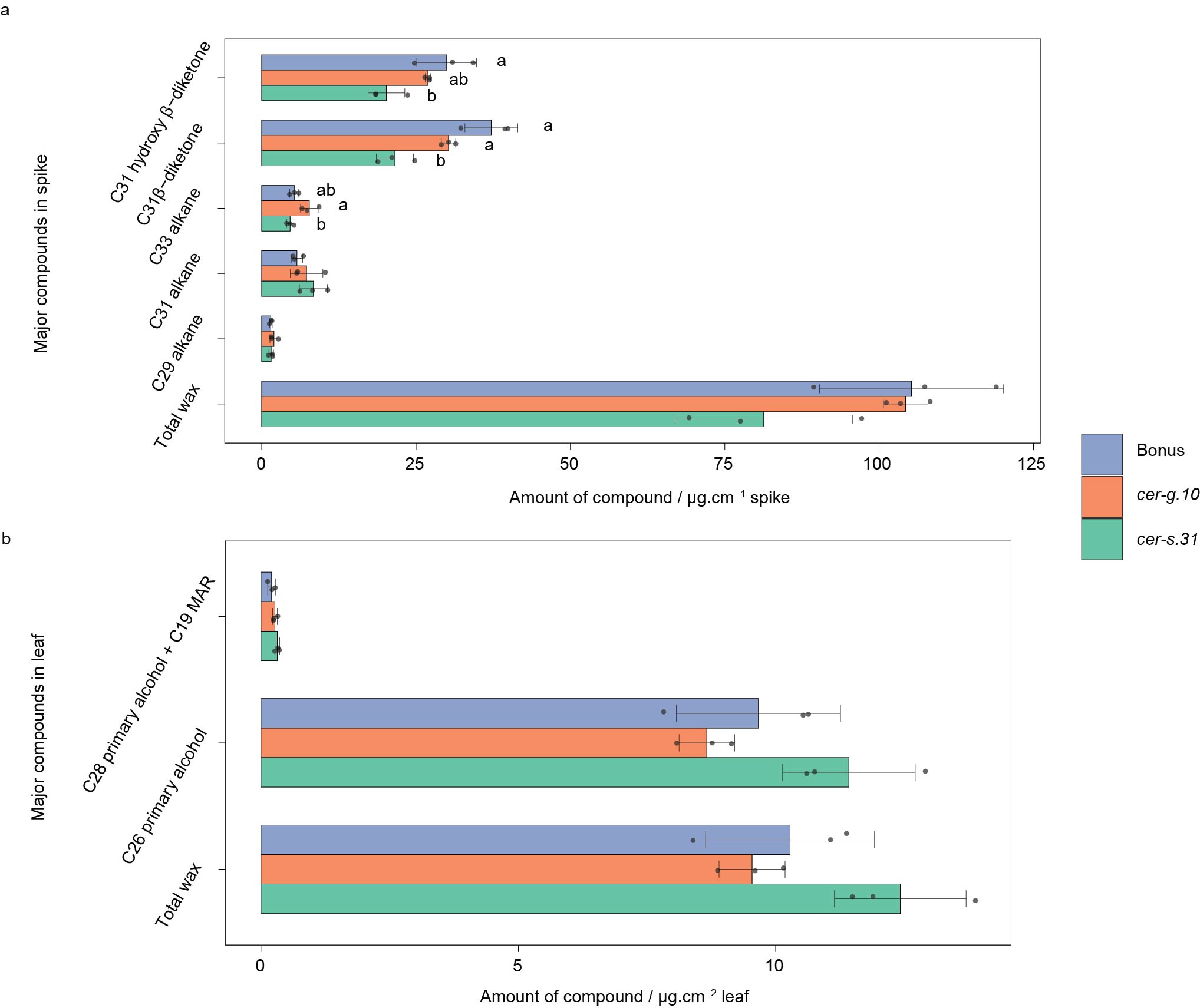


**Supplementary Fig.2. Major wax components and total wax amounts identified in Bonus, *cer-g.10* and *cer-s.31*. a**, spike minus awns and **b**, leaf. Dots represent individual bio-replicates while histograms represent the means ± standard deviation of abundance per leaf area (μg cm^‑2^) or spike length (with awns removed, μg cm^‑1^). N=3 plants per genotype. Different letters indicate significant difference between genotypes (P < 0.05; Tukey’s HSD multiple comparison following one-way ANOVA). Source data including p values of statistic tests are provided in Source data file.


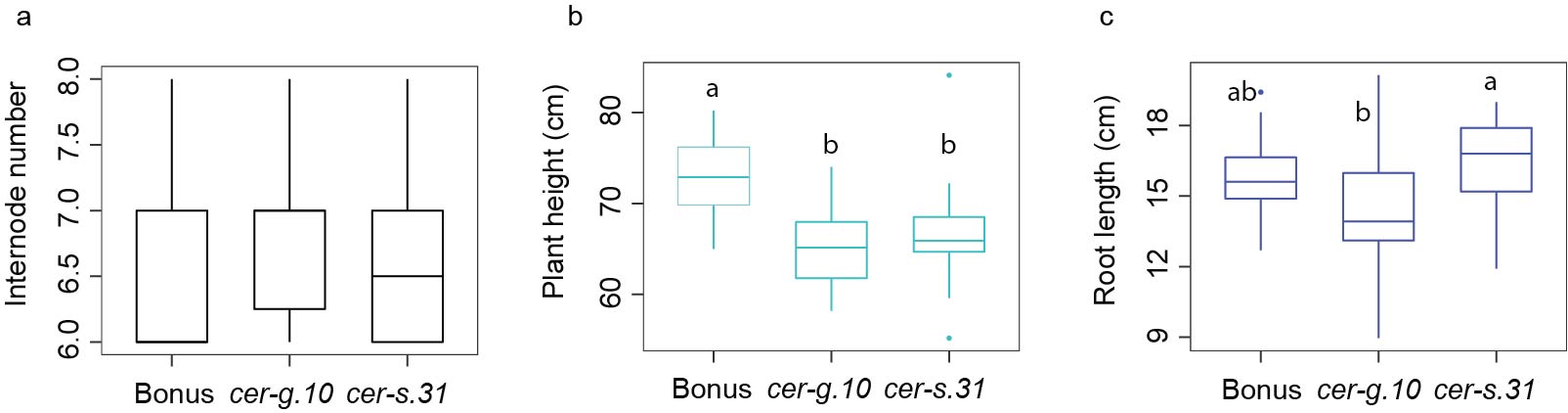


**Supplementary Fig.3. Plant architecture traits of Bonus, *cer-g.10* and *cer-s.31***. **a**, internode number at maturity. N = 10 per genotype. **b**, mature plant height of all genotypes. N=10 per genotype. **c**, mean lengths of longest five roots at maturity. N = 22 per genotype. In each box plot, the lower and upper box edges represent the first and third quartiles, the horizontal lines indicate the median, and the lower and upper whiskers denote the minimal and maximal values within 1.5* interquartile range, respectively, while points indicate outliers beyond this range. Different letters indicate significant difference (p < 0.05; Dunn’s test following a Kruskal-Wallis test) between genotypes. Source data including p values of statistic tests are provided in Source data file.


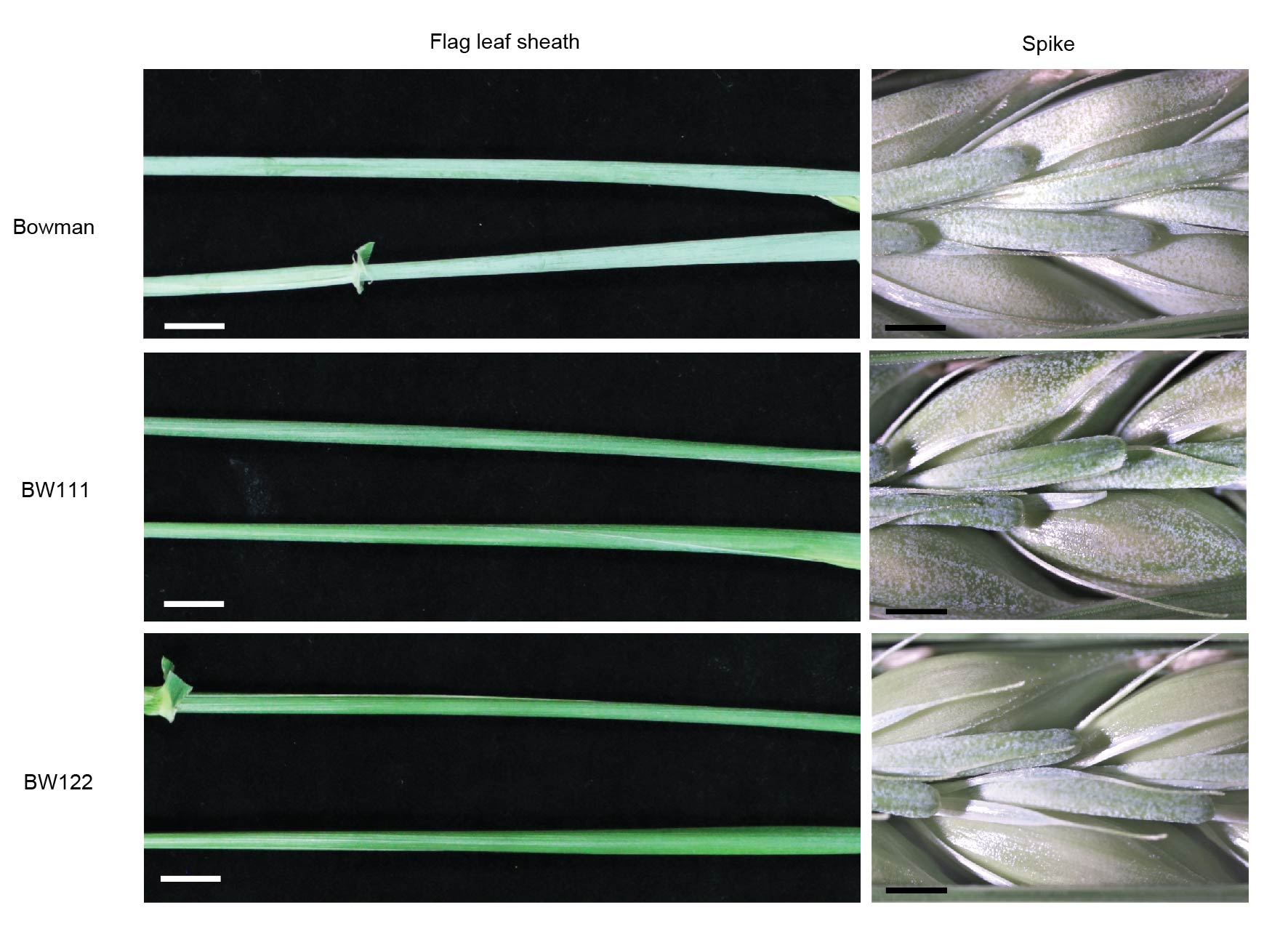


**Supplementary Fig.4. Confirmation of mutant phenotypes in the Bowman near-isogenic lines (BwNILs).** BwNILs of *cer-g.10* (BW111) and *cer-s.31* (BW122) phenocopy the original mutant wax deficiency on flag leaf sheaths (scale bars = 1 cm) and spikes (scale bars = 2 mm).


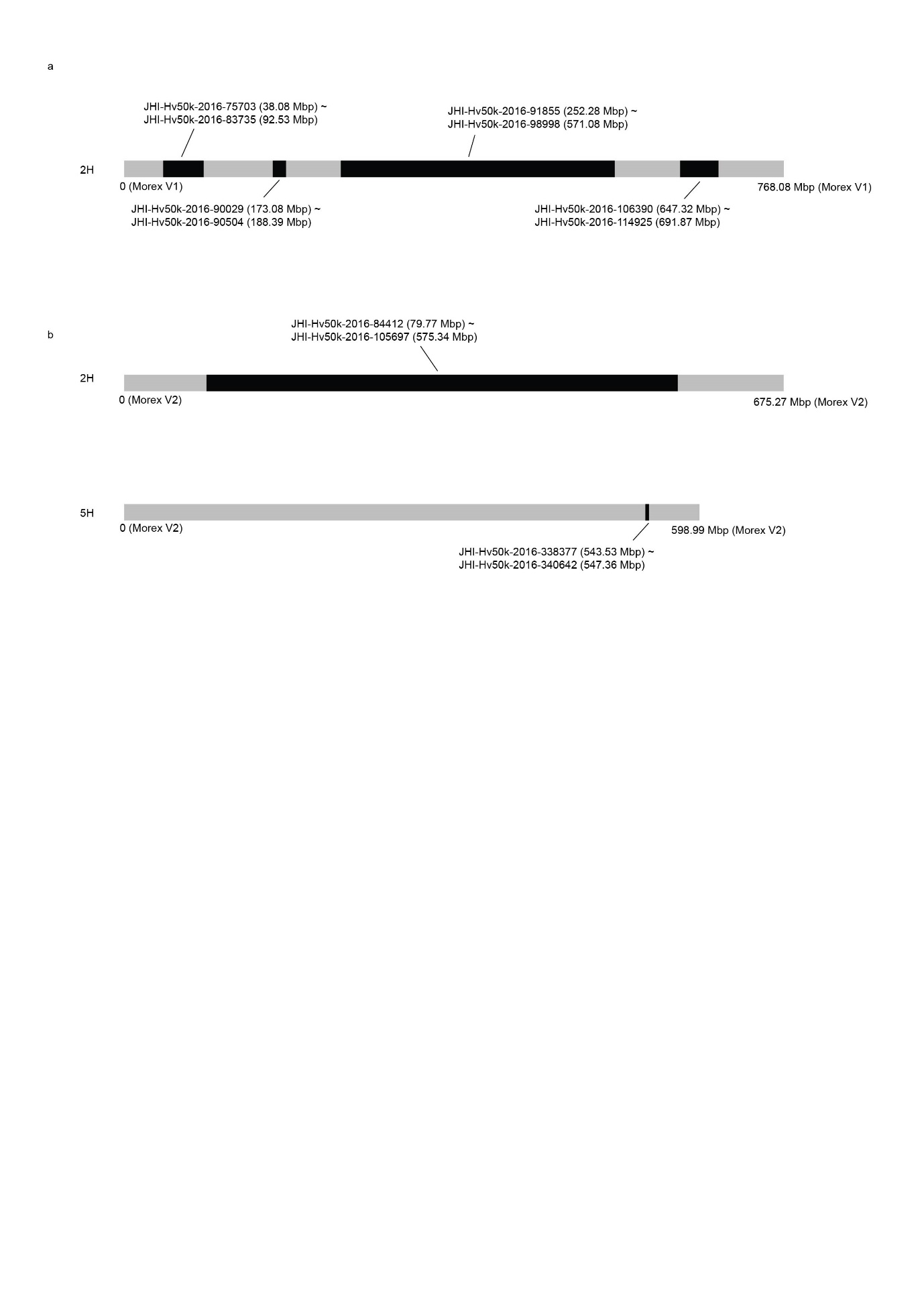


**Supplementary Fig.5. Bonus introgressions in BW111 and BW122 identified using 50K genotyping.** **a**, BW111 introgressions on chromosome 2H. Coordinates based on Morex V1 assembly ^10^. **b**, BW122 introgressions on chromosomes 2H and 5H. Barley 50k iSelect SNP Array ^9^ markers at the start and end of each introgression indicated above and below. Coordinates of makers are based on Morex V2 assembly ^11^.


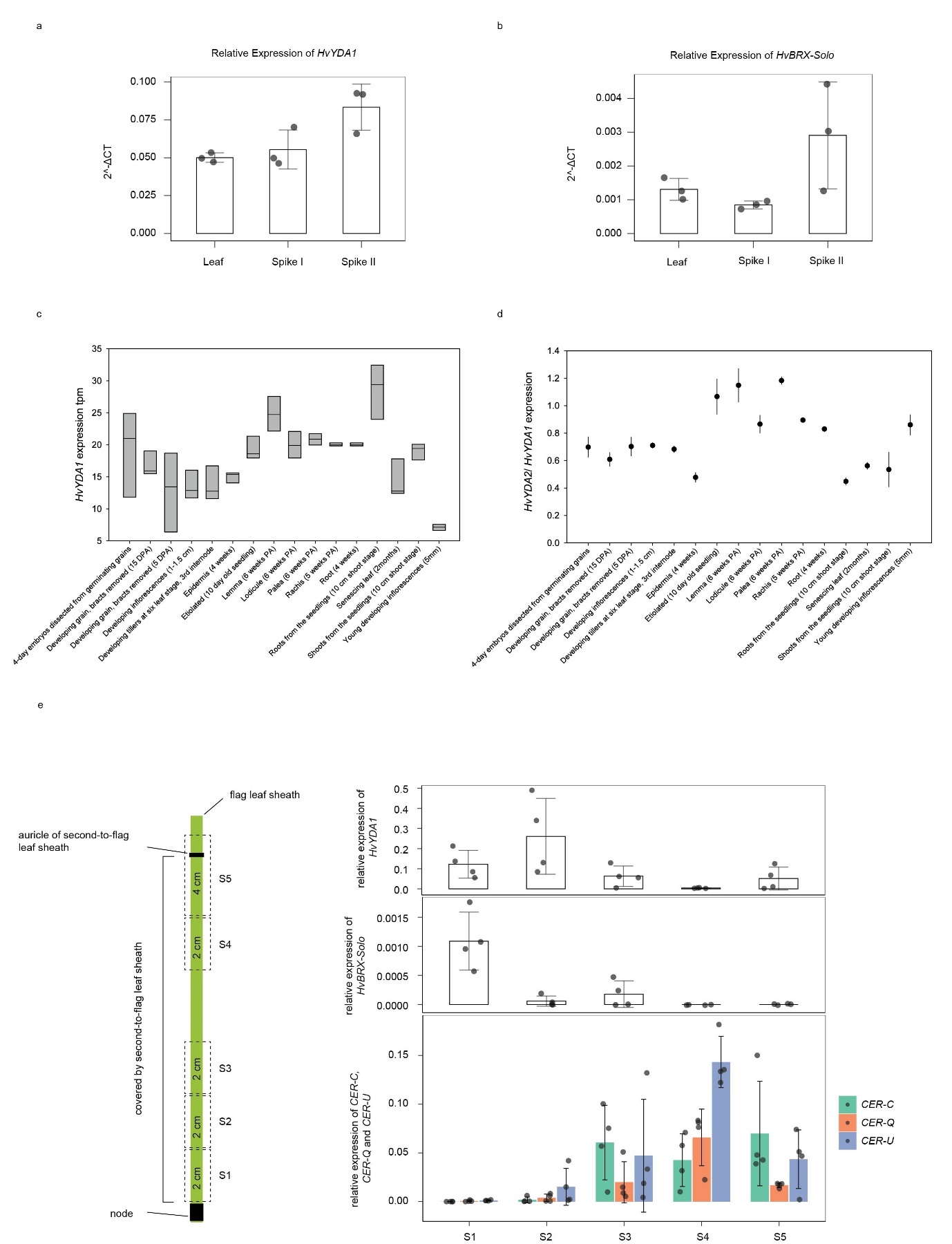


**Supplementary Fig.6. Expression of *HvYDA1/2* and *HvBRX-Solo*.** **a,** *HvYDA1* and **b**, *HvBRX-Solo* in cv. Bowman second leaf 10 days post germination (dpg) and spikes at 29dpg (Spike I) and 35dpg (Spike II, heading stage). Expression is relative to *HvActin7* levels. Dots represent individual bioreplicates while mean ± standard deviation represented in the histogram. **c**, expression of *HvYDA1* in transcripts per million (TPM) derived from the Barley Expression Database (EORNA*; <https://ics.hutton.ac.uk/eorna/index.html>). **d**, ratio of *HvYDA2* and *HvYDA1* expression in vegetative tissues from data presented in (**c**). For box plots, the lower and upper box edges represent the first and third quartiles, the red lines indicate the median, black lines indicate the mean and whiskers show the minimal and maximal values within 1.5* interquartile range, respectively. **e**, illustration of sectioning of flag leaf sheaths of Bowman plants (left) and expression of *HvYDA1, HvBRX-Solo* and *CER-CQU* gene cluster relative to *HvActin7* in these sections. Dots represent individual bioreplicates while mean ± standard deviation represented in the histogram. N= 3 bioreplicates in **a-d** while 4 bioreplicates in **e**. Source data are provided in Source data file.


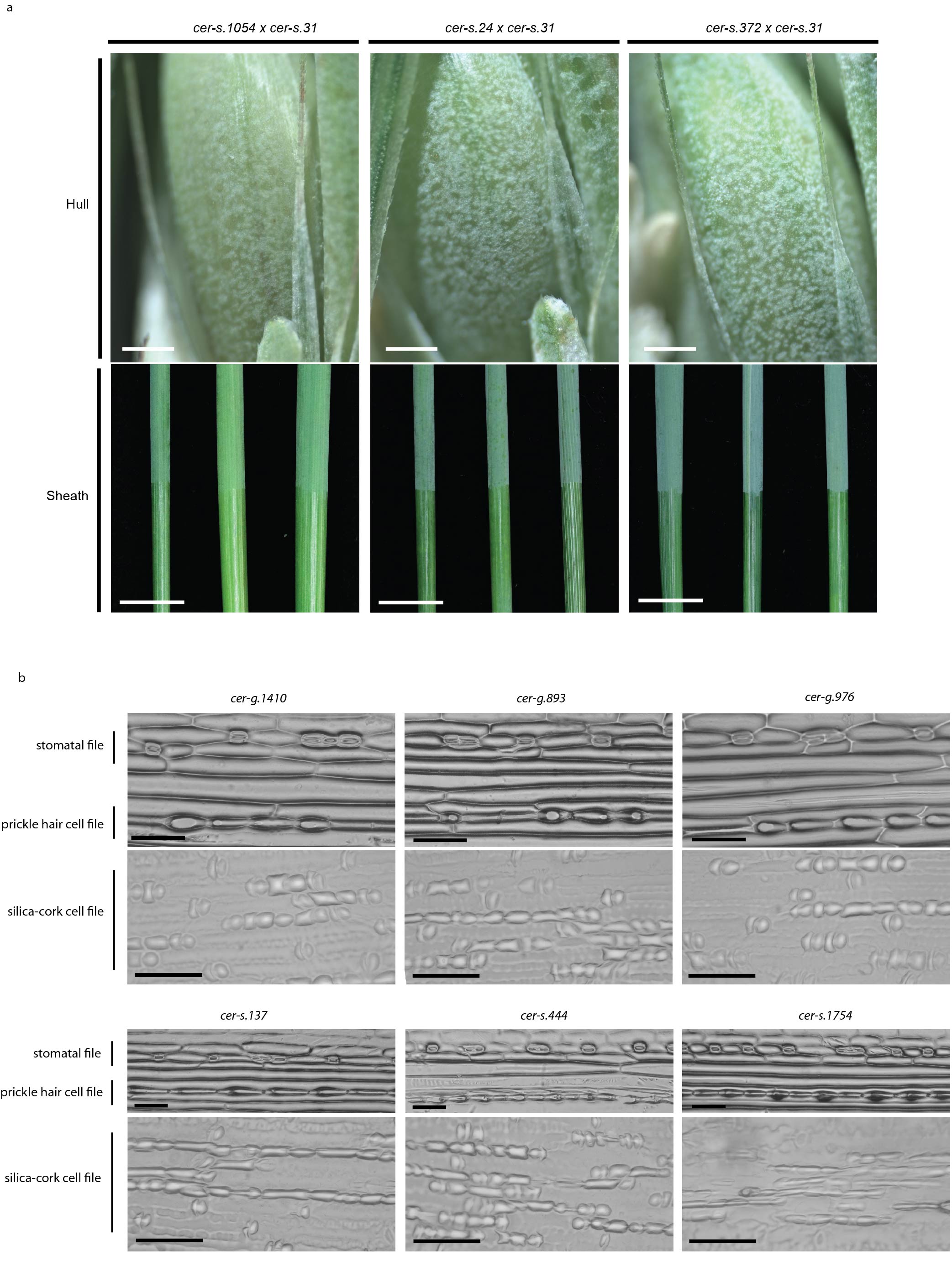


**Supplementary Fig.7: Epidermal patterning of *cer-g* and *cer-s* alleles. a**, visual wax deposition of F1 plants of *cer-s.1054 x cer-s.31*, *cer-s.24 x cer-s.31* and *cer-s.372 x cer-s.31* crossed lines. All lines show evenly distributed wax on lemma (scale bars = 1 mm) and leaf sheath (wax on the bottom half is manually removed for visual contrast. scale bars = 1 cm) surfaces. **b**, stomatal and prickle hair cell patterning in the second leaves (scale bars = 100 µm) and silica-cork cell patterning in flag leaf sheaths (scale bars = 50 µm) of *cer-g* and *cer-s* alleles with premature stop codons. N = 4 plants per genotype.


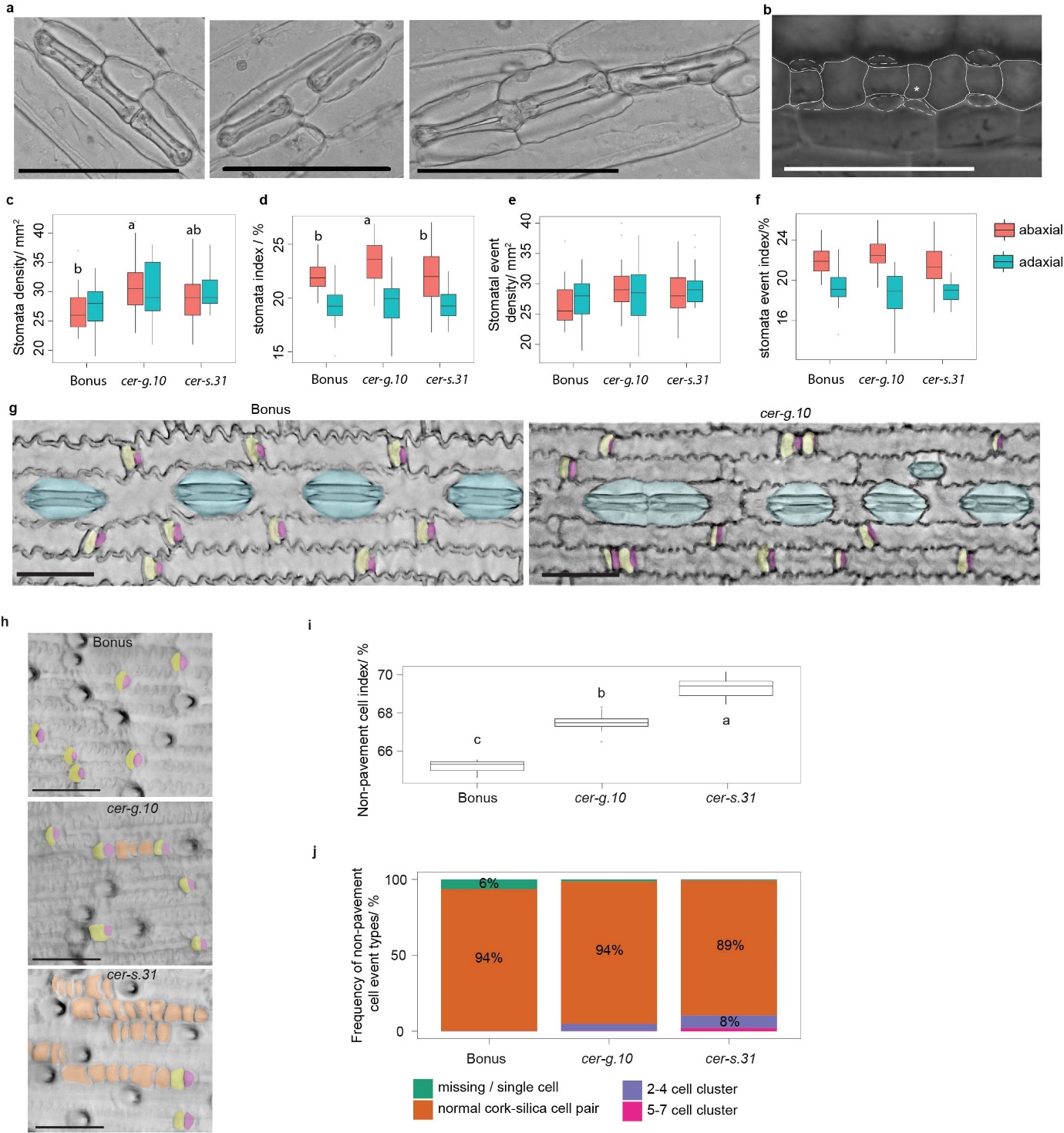


**Supplementary Fig.8. Epidermal cell development in Bonus, *cer-s.31* and *cer-g.10*. a-g,** stomatal complex development. **a**, representative images of stomatal pattering abnormality in the first leaves. From left: triple cluster of stomata, stomata with misshapen subsidiary cells and stomatal cluster with abnormally divided guard cells in “C” shape. Scale bars = 100 µm**.** N = 24 plants for each cell event. **b**, abnormal stomatal development in leaves prior to emergence of 3 day(d)-old seedings of *cer-g.10*. Solid lines outline stomatal lineage ground cells (SLGCs) and guard mother cells (GMCs) while dashed lines indicate developing subsidiary cells. *Asterisk highlights a supernumerary cell which may have GMC identity with a flanking subsidiary cell recruited below. Scale bar = 50 µm. N = 6 plants per genotype**.** **c-f**, stomatal (event) density and index on abaxial and adaxial sides of the first leaf of 14d-old plants. N=24 plants per genotype. Different letters indicate significant difference (P < 0.05; Tukey’s HSD multiple comparison following one-way ANOVA). **g**, representative images of stomatal (blue) and silica (pink)-cork (yellow) cell patterning in flag leaf sheaths of Bonus and *cer-g.10* mutants. Scale bars = 50 µm. N = 8 plants per genotype**. h-j**, silica-cork cell patterning. **h**, representative images of silica–cork cell patterning present on the adaxial lemma of Bonus, *cer-g.10* and *cer-s.31* heading stage plants. Bonus only produces normal silica (pink)–cork (yellow) cell pair while *cer-g.10* and *cer-s.31* present clustered arrays of silica–cork cell pairs and/or cells with unclear identity (orange). Scale bars = 100 µm. N = 8 plants per genotype. **i**, cell index showing the ratio of non-pavement epidermal cells (including silica–cork cell pairs and cells with unclear identity) to total cells in silica–cork cell files in non-costal (not over vasculature) files of the abaxial leaf sheath of Bonus and the *cer-g.10* and *cer-s.31* mutants. Different letters indicate significant difference (p < 0.05; Dunn’s test following a Kruskal-Wallis test) between genotypes. N = 8 plants per genotype. **j**, stacked bar graphs showing frequencies of normal silica–cork cell pair and abnormal cell patterning (missing or single silica cell, cluster of two to four and five to seven silica–cork or unclear identity cells) events in non-costal files Bonus and the *cer-g.10* and *cer-s*.*31* mutants. N = 8 plants per genotype. In each boxplot, the lower and upper box edges represent the first and third quartiles, the horizontal lines indicate the median, and the lower and upper whiskers denote the minimal and maximal values within 1.5* interquartile range, respectively, while points indicate outliers beyond this range. Source data including p values of statistic tests of **c - f, i** and **j** are provided in Source data file.


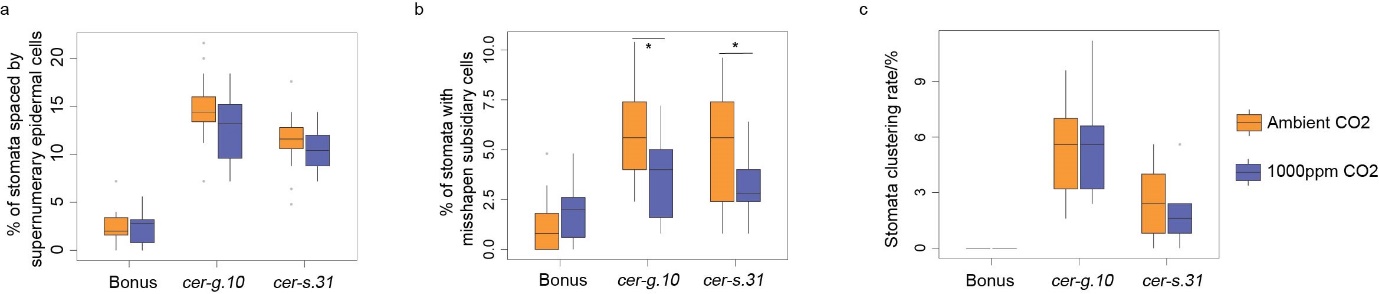


**Supplementary Fig.9. Stomatal development in response to CO_2_ levels.** **a-c**, stomatal development in the abaxial side of first true leaf of 14d-old plants in response to elevated CO_2_ concentration.**a-c**, percentage of stomata intervened by two or more epidermal cells (**a**), stomata with misshapen subsidiary (**b**) and clustered stomata (**c**) within all stomatal events. N=12 plants per genotype per environmental condition. In each box plot, the lower and upper box edges represent the first and third quartiles, the horizontal lines indicate the median, and the lower and upper whiskers denote the minimal and maximal values within 1.5* interquartile range, respectively, while points indicate outliers beyond this range. Asterisks (*) indicate a significant difference (P < 0.05, two-sided *t*-test; df = 22) between control and treatment. Source data including p values of statistic tests are provided in Source data file.


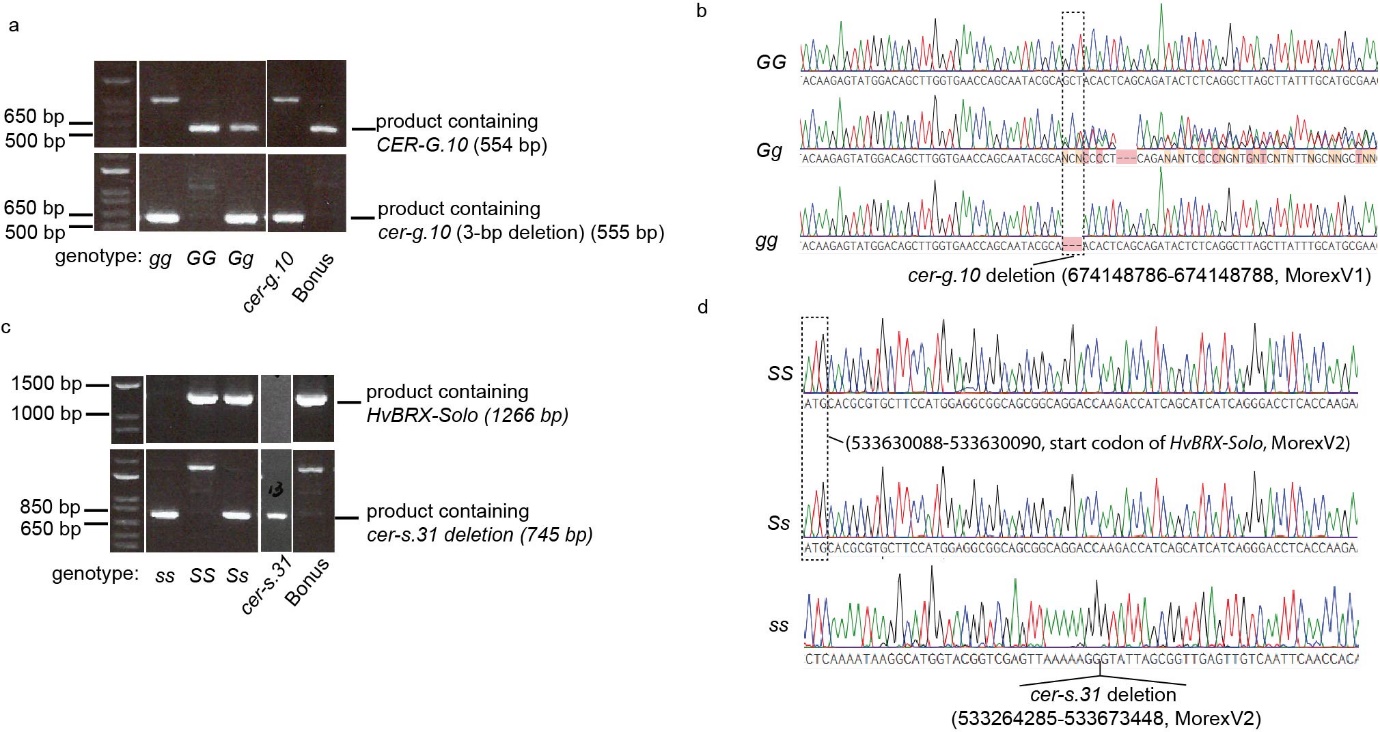


**Supplementary Fig.10. Genotyping of *cer-g.10* and *cer-s.31* loci. a,** representative genotypes of *cer-g.10* locus as represented by electrophoresis gel diagrams of PCR products amplified using genotyping markers (Supplementary Data 15). Two independent genotyping experiments were done for each locus of each sample used for epidermal phenotyping. **b**, sequencing results of +/+, *cer-g.10*/+ and *cer-g.10*/*cer-g.10* at *cer-g.10* locus. **c,** representative genotypes of *cer-s.31* locus as represented by electrophoresis gel diagrams of PCR products amplified using genotyping markers (Supplementary Data 15). **d**, sequencing results of +/+, *cer-s.31*/+ and *cer-s.31*/*cer-s.31* at *cer-s.31* locus. Source data of **a** are provided in Source data file.


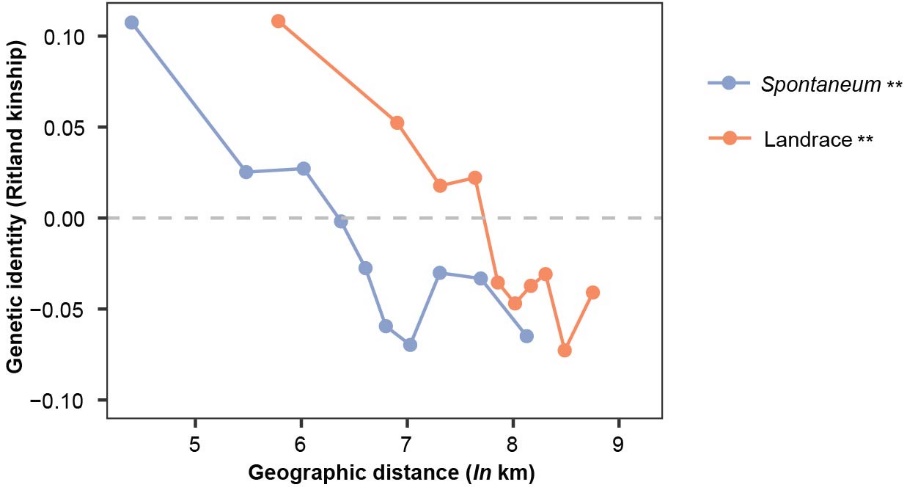


**Supplementary Fig.11. Spatial autocorrelation analysis of exonic SNPs for *HvYDA1* in wild barleys (*Hordeum spontaneum*) and barley landraces (*Hordeum vulgare*).** **P < 0.01 indicates highly significant overall geographic structuring of *HvYDA1* alleles in corresponding barley groups (two-sided permutation test). Analysis was based on Ritland’s kinship coefficient as a measure of genetic identity over geographical distances. Source data including p values of statistic tests are provided in Source data file.


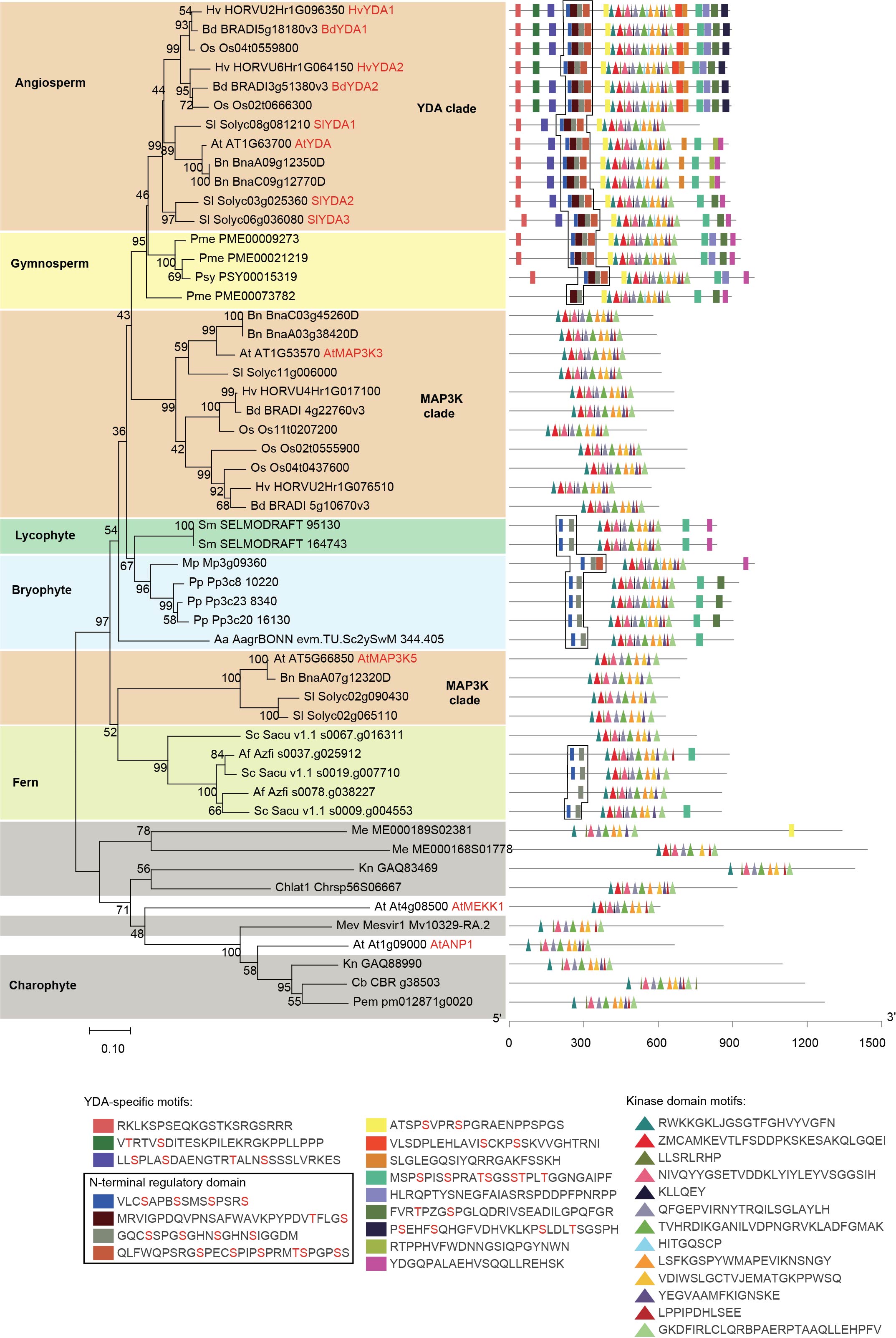


**Supplementary Fig.12. Evolutionary relationship of YDA-like proteins from different species.** Phylogeny (left) and motif conservation (right) of MAPKKK proteins. The phylogenetic tree was inferred using the Neighbour-joining method based on the catalytic domain of HvYDA1 and the putative orthologues above the blast cut-off e-value 1e-115 from other species. The Arabidopsis AtMEKK1 and AtANP1 from adjacent subclades to AtYDA are also included in this tree. Top hit MAPKKKs from six charophyte species with e-values ranging from 1e-74 to 6e-93 are used as the outgroup members to root the tree. Functionally characterised members in the tree are noted by gene names in red. The percentage of trees in which the associated taxa clustered together in 1,000 bootstrap replications is shown next to the branches. The schematic representation of conserved motifs in the YDA-like orthologues was analysed by MEME. Only motifs representing the kinase domain and those specific to the YDA clade members versus the MAPKKK3 and MAPKKK5 clade members of Angiosperm are shown in the schematic diagram. The consensus sequences of each motif are shown at the bottom. The black box denotes motifs in the YDA auto-inhibitory region (aa184-322 of AtYDA). Scale bar indicates genetic variation for the length of the scales. *Aa, Anthoceros agrestis BONN; At, Arabidopsis thaliana; Af, Azolla filiculoides; Bd, Brachypodium distachyon; Bn, Brassica napus; Cb, Chara braunii; Chla, Chlorokybus atmophyticus; Hv, Hordeum vulgare;* *Kn, Klebsormidium nitens;* *Me, Mesotaenium endlicherianum; Mev, Mesostigma viride*; *Mp, Marchantia polymorpha; Os,* *Oryza sativa Japonica; Pp Physcomitrium patens; Psy, Pinus sylvestris;* *Pem, Penium margaritaceum; Pme, Pseudotsuga menziesii; Sc, Salvinia cucullate;* *Sl, Solanum lycopersicum;* *Sm, Selaginella moellendorffii.*


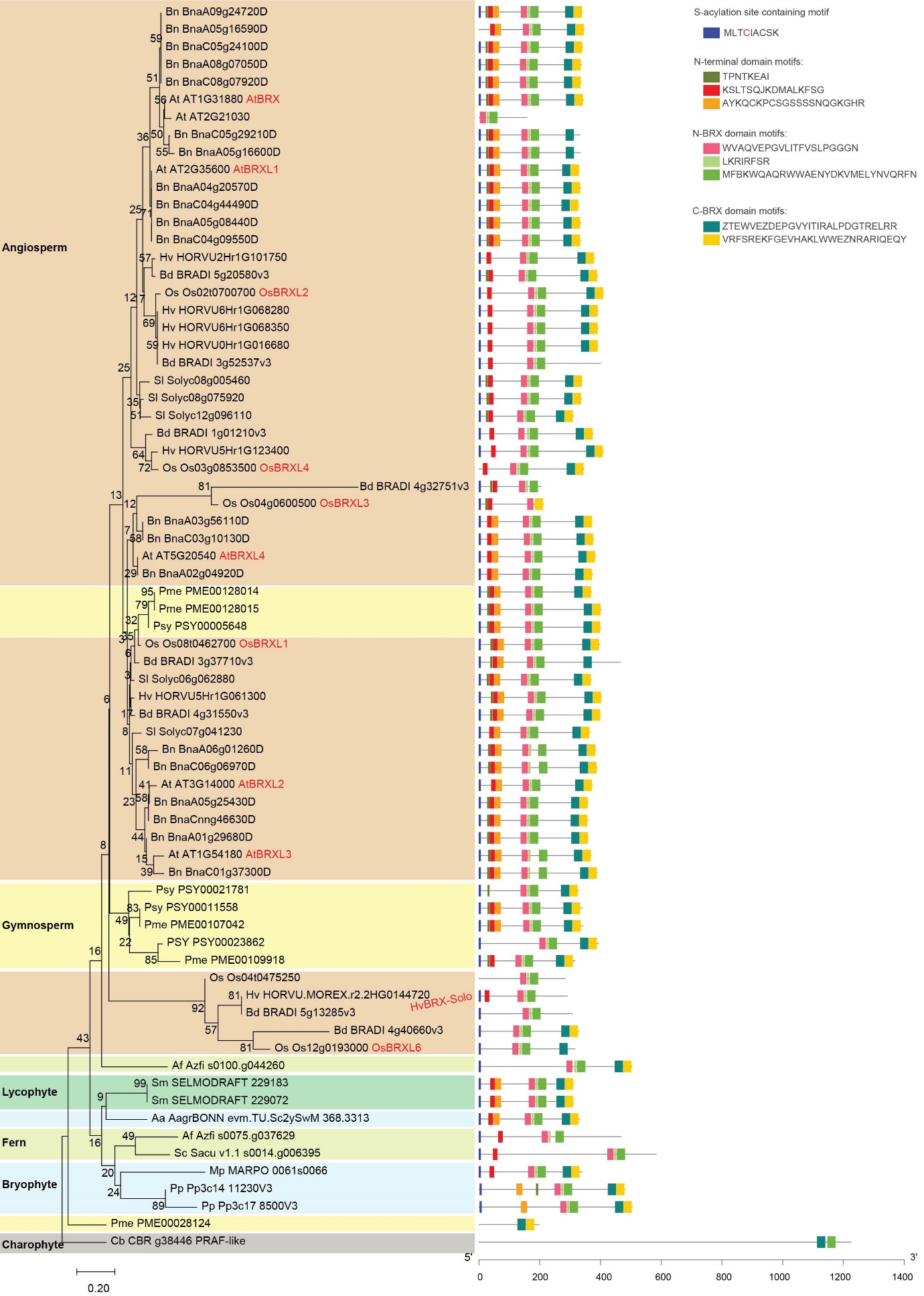


**Supplementary Fig.13. Evolutionary relationship of BRXf proteins from different species.** phylogeny (left) and motif conservation (middle) of BRX-like proteins. The phylogenetic tree was built based on the N-BRX domain in the protein sequence alignment of HvBRX-Solo and its orthologues from other species, using the same inferring and presenting methods as used for the YDA tree. The *Chara braunii* PRAF-like protein that contains the BRX domain was used as the outgroup member to root the tree. Only motifs representing the S-acylation site, N-terminal domain and BRX domains in every protein are displayed. The consensus sequences of each motif are shown on the right, where the putative S-acylation sites are coloured red. Scale bar indicates genetic variation for the length of the scales. *Aa, Anthoceros agrestis BONN; At, Arabidopsis thaliana; Af, Azolla filiculoides; Bd, Brachypodium distachyon; Bn, Brassica napus; Cb, Chara braunii; Chla, Chlorokybus atmophyticus; Hv, Hordeum vulgare;* *Kn, Klebsormidium nitens;* *Me, Mesotaenium endlicherianum; Mev, Mesostigma viride*; *Mp, Marchantia polymorpha; Os,* *Oryza sativa Japonica; Pp Physcomitrium patens; Psy, Pinus sylvestris;* *Pem, Penium margaritaceum; Pme, Pseudotsuga menziesii; Sc, Salvinia cucullate;* *Sl, Solanum lycopersicum;* *Sm, Selaginella moellendorffii*

**Supplementary References**

1 Lundqvist, U. & Franckowiak, J. D. BGS 355, Glossy sheath 5, gsh5. *Barley Genet. Newsl.* **47**, 128–130 (2017).

2 Lundqvist, U. & Franckowiak, J. D. BGS 402, Eceriferum-g, cer-g. *Barley Genet. Newsl.* **47**, 131–132 (2017)

3 Fester, T., and B. Søgaard. The localization of eceriferum loci in barley. *Hereditas* **61**:327-337. (1969)

4 Komatsuda, T., et al. 2007. Six-rowed barley originated from a mutation in a homeodomain-leucine zipper I-class homeobox gene. *Proc Natl Acad Sci*, **104** 1424-1429 (2007)

5 Rasmusson, D.C., and J.W. Lambert. Inheritance of the glossy-sheath character in barley, *Hordeum vulgare*L*. Crop Sci*. **5**:251-253. (1965).

6 Søgaard, B. Linkage studies on eceriferum mutants in barley. BGN **1**:41-47. (1971).

7 Takahashi, R., J. Hayashi, T. Konishi, and I. Moriya. Inheritance and linkage studies in barley. V. Locating seven new mutant genes. Ber. Ohara Inst. landw. Biol., Okayama Univ. **15**:147-168. (1972).

8 Søgaard, B. Continued linkage studies on eceriferum mutants in barley. BGN **3**:57-61. (1973).

9 Bayer, M. M. *et al.* Development and evaluation of a barley 50k iSelect SNP array. *Front. Plant Sci.* doi:10.3389/fpls. (2017)

10 Mascher, M. et al A chromosome conformation capture ordered sequence of the barley genome. *Nature* **544**, 427–433 (2017).

11 Monat, C. *et al.* TRITEX: chromosome-scale sequence assembly of Triticeae genomes with open-source tools. *Genome Biol.* **20**, 284 (2019).
