# Supplementary material for: Conserved signalling components coordinate epidermal patterning and cuticle deposition in barley
Source: Nat Commun. 2022 Oct 13;13:6050. doi: 10.1038/s41467-022-33300-1 (PMC9561702; doi:10.1038/s41467-022-33300-1)
Supplement: Supplementary file 18 — Reporting Summary [file 41467_2022_33300_MOESM18_ESM.pdf]

nature portfolio

Corresponding author(s): Sarah McKim

Last updated by author(s): 26 August 2022

Reporting Summary

Nature Portfolio wishes to improve the reproducibility of the work that we publish. This form provides structure for consistency and transparency in reporting. For further information on Nature Portfolio policies, see our Editorial Policies and the Editorial Policy Checklist.

Statistics

For all statistical analyses, confirm that the following items are present in the figure legend, table legend, main text, or Methods section.

n/a	Confirmed	
☐	☒	The exact sample size (*n*) for each experimental group/condition, given as a discrete number and unit of measurement
☐	☒	A statement on whether measurements were taken from distinct samples or whether the same sample was measured repeatedly
☐	☒	The statistical test(s) used AND whether they are one- or two-sided<br>*Only common tests should be described solely by name; describe more complex techniques in the Methods section.*
☒	☐	A description of all covariates tested
☐	☒	A description of any assumptions or corrections, such as tests of normality and adjustment for multiple comparisons
☐	☒	A full description of the statistical parameters including central tendency (e.g. means) or other basic estimates (e.g. regression coefficient) AND variation (e.g. standard deviation) or associated estimates of uncertainty (e.g. confidence intervals)
☐	☒	For null hypothesis testing, the test statistic (e.g. *F*, *t*, *r*) with confidence intervals, effect sizes, degrees of freedom and *P* value noted<br>*Give P values as exact values whenever suitable.*
☒	☐	For Bayesian analysis, information on the choice of priors and Markov chain Monte Carlo settings
☒	☐	For hierarchical and complex designs, identification of the appropriate level for tests and full reporting of outcomes
☒	☐	Estimates of effect sizes (e.g. Cohen's *d*, Pearson's *r*), indicating how they were calculated

Our web collection on statistics for biologists contains articles on many of the points above.

Software and code

Policy information about availability of computer code

Data collection	1) GC-MS data were collected using the XCalibur software suite Thermo Fisher Scientific). Leaf area and spike length were calculated using ImageJ (NIH).<br>2) Sequencing read quality was assessed using FastQC . A manual review of the mapping data was performed using Tablet. Single-sample SNP calling was performed using the GATK Best Practices Pipeline. The called SNPs were filtered using bcftools (SAMtools) using a minimum base quality of 100 and a minimum read depth of 30 before being visualised.<br>3) Primers were designed using the Web-based Allele Specific Primer (WASP) tool.<br>4) Genomic DNA from BW122, Bowman and Bonus were subjected to whole-genome sequencing on the Illumina MiSeq platform.<br>5) For the RNAseq, raw data were mapped to the Barley Reference Transcript BaRTv1.0 90 for quantification using Samtools.<br>6) Guard cell lengths were measured in Volocity (Quorum Technologies).<br>7) A strand-specific mRNA library was constructed and then sequenced on a NovaSeq 6000 PE150 platform at Novogene UK with 2 × 40 million 150-bp paired-end reads for each replicate.
Data analysis	1) Mapping Exome capture and other mapping data were visualised using R. Read quality was assessed using FastQC. Reads were mapped with BWA version 0.7.17 A manual review of the mapping data was performed using Tablet. Single-sample SNP calling was performed using the GATK Best Practices Pipeline. The called SNPs were filtered using bcftools (SAMtools) using a minimum base quality of 100 and a minimum read depth of 30 before being visualised. KASP primers sets. KASP primers were designed using the Web-based Allele Specific Primer (WASP) tool. Raw reads were mapped to the Morex v2 assembly with BWA. Sambamba was used to remove duplicate reads and reads with more than six mismatches. Mapping data was manually reviewed using Tablet. The GATK toolkit was used to re-align indels and recalibrate variant calls, producing a recalibrated BAM file. Small variant calling of BW122, Bowman and Bonus was performed using freebayes and structural variant calling was performed using Manta SV caller.<br>2) Raw RNA-seq data were mapped to the Barley Reference Transcript BaRTv1.0 for quantification using Samtools. A differential expression

analysis was performed using 3D RNA-seq App. The enriched GO terms were summarised using ReviGo.
3) Lengths of guard cells were imaged using Volocity from Quorum Technologies.
4) Joining haplotype network construction was performed using PopArt.
5) Spatial auto-correlation analysis was undertaken using SPAGeDi. Pairwise geographical distances between accessions were calculated using the R package "geodesic". Finally, landraces with geographic co-ordinates were plotted on a world map using the R package "rworldmap.
6) Phylogenetic trees were constructed using MEGA X with sequence alignment performed using ClustalW and the trees inferred using the neighbour-joining method.
7) Conserved motifs among the proteins were explored using the MEME motif search tool. Resulting motif structure of the proteins were aligned with the phylogenetic trees using TBtools
8) All statistical analyses were carried out using R.

For manuscripts utilizing custom algorithms or software that are central to the research but not yet described in published literature, software must be made available to editors and reviewers. We strongly encourage code deposition in a community repository (e.g. GitHub). See the Nature Portfolio guidelines for submitting code & software for further information.

Data

Policy information about availability of data

All manuscripts must include a data availability statement. This statement should provide the following information, where applicable:

- Accession codes, unique identifiers, or web links for publicly available datasets
- A description of any restrictions on data availability
- For clinical datasets or third party data, please ensure that the statement adheres to our policy

All materials described in the paper will be made freely available. Source data are provided with this paper. RNA-Seq and exome capture data sets are deposited into the European Nucleotide Archive (ENA), http://www.ebi.ac.uk/ena, under this project accession number PRJEB53837 [https://www.ebi.ac.uk/ena/browser/view/PRJEB53837]

Human research participants

Policy information about studies involving human research participants and Sex and Gender in Research.

Reporting on sex and gender	*Use the terms sex (biological attribute) and gender (shaped by social and cultural circumstances) carefully in order to avoid confusing both terms. Indicate if findings apply to only one sex or gender; describe whether sex and gender were considered in study design whether sex and/or gender was determined based on self-reporting or assigned and methods used. Provide in the source data disaggregated sex and gender data where this information has been collected, and consent has been obtained for sharing of individual-level data; provide overall numbers in this Reporting Summary. Please state if this information has not been collected. Report sex- and gender-based analyses where performed, justify reasons for lack of sex- and gender-based analysis.*
Population characteristics	*Describe the covariate-relevant population characteristics of the human research participants (e.g. age, genotypic information, past and current diagnosis and treatment categories). If you filled out the behavioural & social sciences study design questions and have nothing to add here, write "See above."*
Recruitment	*Describe how participants were recruited. Outline any potential self-selection bias or other biases that may be present and how these are likely to impact results.*
Ethics oversight	*Identify the organization(s) that approved the study protocol.*

Note that full information on the approval of the study protocol must also be provided in the manuscript.

Field-specific reporting

Please select the one below that is the best fit for your research. If you are not sure, read the appropriate sections before making your selection.

☒ Life sciences ☐ Behavioural & social sciences ☐ Ecological, evolutionary & environmental sciences

For a reference copy of the document with all sections, see nature.com/documents/nr-reporting-summary-flat.pdf

Life sciences study design

All studies must disclose on these points even when the disclosure is negative.

Sample size	Sample sizes were chosen to maximise independent biological replicates while balancing this consideration with reasonable growth space and adequate handling time. Sample sizes for phenotyping measurements were typically from ten individuals or greater, which we feel captures individual within genotype variation, as demonstrated by the small sample error estimates. The four RNAseq samples each represented ten pooled individuals, which showed good clustering by PCA. qRT-PCR represented three biological replicates, each consisting of ten pooled individuals; given that n=3, we represent individual data points from each replicate in our data. We also represent individual bioreplicates (N = 3) as dots for the GC-MS values in Supplementary Figure 2.

Data exclusions	Poor quality SNP genotyping calls and RNA-seq gene expression below reliable detection thresholds were excluded, as is common practice in both types of analyses.
Replication	All experiments included independent biological replication of at least n =3.
Randomization	Samples were not assigned to random groups. Samples differed by genotype.
Blinding	blinding was not relevant to our study.

Reporting for specific materials, systems and methods

We require information from authors about some types of materials, experimental systems and methods used in many studies. Here, indicate whether each material, system or method listed is relevant to your study. If you are not sure if a list item applies to your research, read the appropriate section before selecting a response.

Materials & experimental systems

n/a	Involved in the study	
☒	☐	Antibodies
☒	☐	Eukaryotic cell lines
☒	☐	Palaeontology and archaeology
☒	☐	Animals and other organisms
☒	☐	Clinical data
☒	☐	Dual use research of concern

Methods

n/a	Involved in the study	
☒	☐	ChIP-seq
☒	☐	Flow cytometry
☒	☐	MRI-based neuroimaging
